# Adipose Tissue Distribution and Body Mass Index (BMI) Correlation With Daily Image-Guided Radiotherapy (IGRT) Shifts of Abdominal Radiation Therapy Patients

**DOI:** 10.7759/cureus.40979

**Published:** 2023-06-26

**Authors:** Ryan G Price, Shane Lloyd, Xuechen Wang, Ben Haaland, Geoff Nelson, Bill Salter

**Affiliations:** 1 Radiation Oncology, University of Utah School of Medicine; Huntsman Cancer Institute, Salt Lake City, USA; 2 Biostatistics, University of Utah, Salt Lake City, USA; 3 Medical Physics, Radiation Oncology, University of Utah School of Medicine; Huntsman Cancer Institute, Salt Lake City, USA

**Keywords:** abdominal radiation therapy, igrt, setup uncertainty, igrt shifts, body mass index: bmi

## Abstract

Purpose

There are several studies suggesting a correlation between image-guided radiotherapy (IGRT) setup errors and body mass index (BMI). However, abdominal fat content has visceral and subcutaneous components, which may affect setup errors differently. This study aims to analyze a potential workflow for characterizing adipose content and distribution in the region of the target that would allow a quickly calculated metric of abdominal fat content to stratify these patients.

Methods

IGRT shift data was retrospectively tabulated from daily fan-beam CT-on-rails pre-treatment alignment for 50 abdominal radiation therapy (RT) patients, and systematic and random errors in the daily setup were characterized by tabulating average and standard deviations of shift data for each patient and looking at differences for different distributions of adipose content. Visceral and subcutaneous fat content were defined by visceral fat area (VFA) and subcutaneous fat area (SFA) using a region-growing algorithm to contour adipose tissue on CT simulation scans. All contours were created for a single slice at the treatment isocenter, on which the VFA and SFA were calculated. A log-rank test was used to test trends in shifts over quartiles of adiposity.

Results

VFA ranged from 1.9-342.8c m^2^, and SFA from 11.8-756.0 cm^2^. The standard definition (SD) of random error (σ) in the lateral axis for Q1 vs. Q4 VFA was 0.10cm vs. 0.29cm, 0.12cm vs. 0.28cm for SFA, and 0.12cm vs. 0.31cm for BMI. The percentage of longitudinal shifts greater than 10mm for Q1 vs. Q4 VFA was 0% vs. 9%, 2% vs. 19% for SFA, and 0% vs. 20% for BMI. Statistically significant trends in shifts vs. the BMI quartile were seen for both pitch and the longitudinal direction, as well as for pitch corrections vs. the VFA quartile.

Conclusion

Within this dataset, abdominal cancer patients showed statistically significant trends in shift probability vs. BMI and VFA. Also, patients in the upper quartiles of all adiposity metrics showed an increased SD of σ in the lateral direction and increased shifts over 10 mm in the longitudinal direction. However, despite these relationships, neither VFA nor SFA offered discernible advantages in their relationship to shift uncertainty relative to BMI.

## Introduction

Patient obesity poses many challenges in the context of radiation therapy (RT) and cancer care in general. There have been a number of studies showing that obesity could be related to inferior oncologic outcomes for various types of cancer and treatment modalities [1-6]. Additionally, several studies have shown a potential correlation between obesity and an increased risk of clinical recurrence and biochemical failure in prostate cancer when utilizing external beam radiation therapy (EBRT) [7-9]. However, there are also many studies that fail to show body mass index (BMI) itself as an independent predictor of outcomes [10-12].

There have been some studies showing that in the context of RT, body mass index (BMI) may be correlated with increased setup variability and uncertainty, indicating that decisions about image guidance and margins might need to be made with the BMI of the patient in mind [13-15]. However, while BMI is a widely accepted measure of obesity, there can be quite a bit of variability in how that fatty tissue is distributed for various patients of similar BMI [16,17]. As an alternative to BMI, several studies have utilized the high quality of current computed-tomography (CT) imaging to characterize patients by the amount of visceral fat content and subcutaneous fat content in the scan [3,16-18], and have found this metric to be more strongly correlated with outcomes than BMI alone [3,18]. The purpose of this study was to utilize retrospective CT imaging and shift data for abdominal cancer patients to characterize the distribution of fat content in the abdomen and its possible correlation with setup uncertainty.

## Materials and methods

Patient and treatment characteristics

With approval from the institutional review board of the University of Utah (IRB 00048188), 50 patients who had previously received radiation therapy for cancer in the abdomen were retrospectively reviewed. This consisted of 25 patients with liver cancer, 13 patients with pancreatic cancer, eight stomach cancer patients, two being treated for abdominal lymph nodes, one for adrenal cancer, and one for bile duct treatment. Patients were treated with a range of fractionation patterns and received anywhere from three to 30 fractions of RT, resulting in 809 total reviewed fractions. All patient-recorded shifts were the clinically utilized shifts from the initial setup position as determined by Siemens Sensation Open (Siemens, Concord, CA) CT-On-Rails (CTOR). BMI was collected for all patients near the time of simulation and ranged between 18 and 60.8 kg/m2.

Most patients were immobilized with the use of a T-vac-lock bag (Elekta, Stockholm, Sweden) or Alpha Cradle (Smithers, North Canton, OH) with an accompanying knee sponge, but for eight of the cases (usually stereotactic body radiotherapy (SBRT)), large body fix bags (Elekta, Stockholm, Sweden) were used. Also, many patients had tumor motion characterization at the time of sim via 4D-CT that was utilized for internal target volume (ITV) target generation, and those who demonstrated unacceptable magnitudes of tumor motion (5 mm-7 mm) were managed with compression techniques. No breath-hold techniques were utilized for any of the included patients. When possible, alignment to a visible soft tissue gross tumor volume (GTV) was preferable; however, some patients required the use of implanted fiducial markers.

Determination of adipose distribution

To quantify the adiposity of each patient, we wanted a measure that could distinguish adipose distribution in the relevant region of IGRT while also not being overly cumbersome for day-to-day use, thus we used a similar metric as described in several other studies as visceral fat area (VFA) and subcutaneous fat area (SFA) [3,5,6,17,19,20]. These metrics are defined as the contoured area of both visceral fat and subcutaneous fat at a defined longitudinal position within the body. Most studies either measure this area at anatomical landmarks such as the umbilicus, L4-L5 vertebral bodies, or L2-L3 vertebral bodies. Given that the longitudinal location of the umbilicus can vary wildly, especially for larger habitus patients, we characterized both VFA and SFA at the location of the treatment isocenter with the hypothesis that adiposity near the site of treatment might correlate more strongly with setup variability. Treatment planning scans were taken either on the Siemens Somatom Confidence (Erlangen, Germany) or on the GE Lightspeed (Erlangen, Germany) with a 2 mm slice thickness, and all adipose contours were performed in MIM (Cleveland, OH) on these scans with MIM’s commercial region growing algorithm and edited manually to correct for obvious contour errors.

Statistical methods

Due to the sparsity of data outside this range, only patients with BMI, VFA, and SFA (n=44) were included in the statistical analyses. Patient adipose measures (BMI, VFA, and SFA) were categorized into quartiles to examine trends in shift magnitude. A log-rank test was used to test for trends in shifts over quartiles of adiposity measures. Generalized estimating equation models were applied to investigate the association between shift magnitude and adipose measures. The same analyses were performed for the subgroup of patients with liver cancer (n=20). Statistical analyses were conducted using R statistical software, version 4.1.1.

Lastly, shift data was used to analyze differences in random setup error using methods as described in Van Herk et al. [21], where the standard deviation in random setup error is defined as the root mean squared of the shift standard deviations for individual patients.

## Results

The distribution of patient data for both the entire dataset and for the largest patient subgroup is summarized in Tables 1-2. On average across the entire dataset, mean errors were 1 mm in the lateral direction, 3 mm in the longitudinal direction, and 2 mm in the vertical, with mean rotational errors of 0.2°, 0.7°, and 0.0° in the yaw, pitch, and roll axes, respectively.

**Table 1:** Summary of patient data LN: lymph node; BMI: body mass index; IQR: interquartile range; VFA: visceral fat area; SFA subcutaneous fat area

Variable	Summary (N = 50)
Treatment site	
Abdominal LN	2 (4%)
Adrenal	1 (2%)
Bile ducts	1 (2%)
Liver	24 (48%)
Liver LN	1 (2%)
Pancreas	13 (26%)
Stomach	6 (12%)
Stomach/esophagus	2 (4%)
BMI: median (IQR; range)	26.5 (23.1 – 30.5; 18 – 60.8)
VFA: median (IQR; Range)	66.0 (19.0 – 123.3; 1.9 – 342.8)
SFA: median (IQR; Range)	128.8 (77.0 – 158.9; 11.8 – 756.0)

**Table 2:** Summary of patient data: treatment site of the liver BMI: body mass index; IQR: interquartile range; VFA: visceral fat area; SFA subcutaneous fat area

Variable	Summary (N = 24)
BMI: median (IQR; range)	27.6 (24.1 – 30.1; 20.8 – 42.6)
VFA: median (IQR; range)	84.2 (20.4 – 138.8; 4.3 – 262.2)
SFA: median (IQR; range)	124 (85 – 154; 12 – 565)

**Figure 1:**
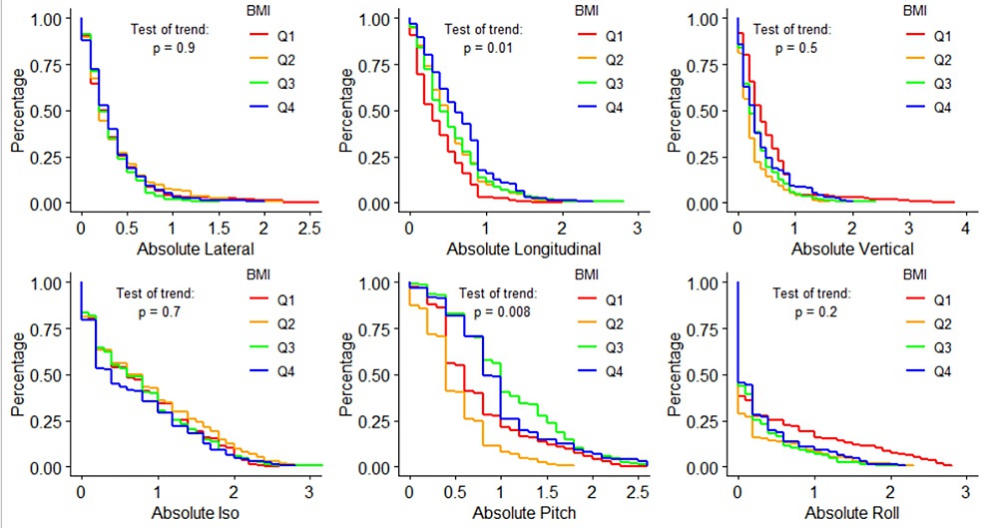
Shift probability curves for each shift direction and each BMI quartile, and the corresponding log-rank results testing for trends in shifts over different BMI quartiles BMI: body mass index; Iso: yaw

Figure 1 visualizes the log-rank test for each shift direction (translational and rotational) over the four BMI quartiles. Q1 of BMI is BMI $\leq$23, Q2 is 23<BMI$\leq$26, Q3 is 26<BMI$\leq$30, Q4 is BMI>30. What these plots fundamentally show is the difference in shift probability for each size or direction of shift and whether the difference in these curves from Q1 through Q4 statistically supports a related trend. For all six tested shifts, both longitudinal and pitch showed statistically significant trends in shift magnitude over the four quartiles, with p-values of 0.01 for absolute (independent of direction) longitudinal shifts and 0.008 for absolute pitch. This data shows frequencies of longitudinal shifts greater than 10 mm as 0.5%, 7.0%, 11.7%, and 19.9% for each quartile, respectively.

**Figure 2:**
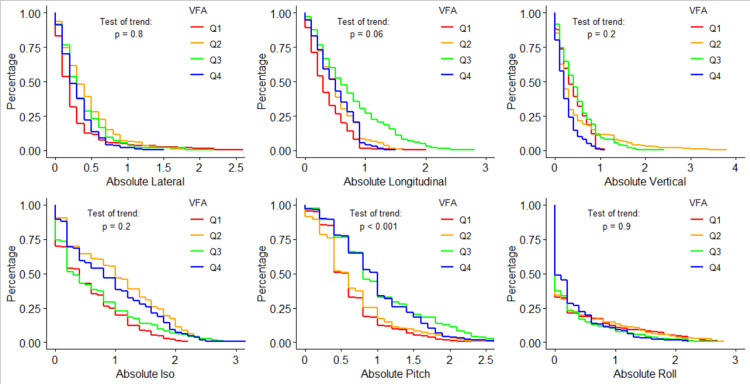
Shift probability curves for each shift direction and each VFA quartile, and the corresponding log-rank results testing for trends in shifts over different VFA quartiles VFA: visceral fat area; Iso: yaw

Figure 2 similarly visualizes the log-rank test for each shift direction, but instead over the four VFA quartiles. Q1 of VFA is VFA$\leq$20 , Q2 is 20<VFA$\leq$65, Q3 is 65<VFA$\leq$120, and Q4 is VFA>120. For all six tested shifts, only pitch showed statistically significant trends in shift magnitude over the four quartiles with a p-value <0.001, though longitudinal shifts fell just below the cutoff with a p-value of 0.06. This data shows frequencies of longitudinal shifts greater than 10 mm as 0.0%, 7.1%, 29.75%, and 8.8% for each quartile, respectively.

**Figure 3:**
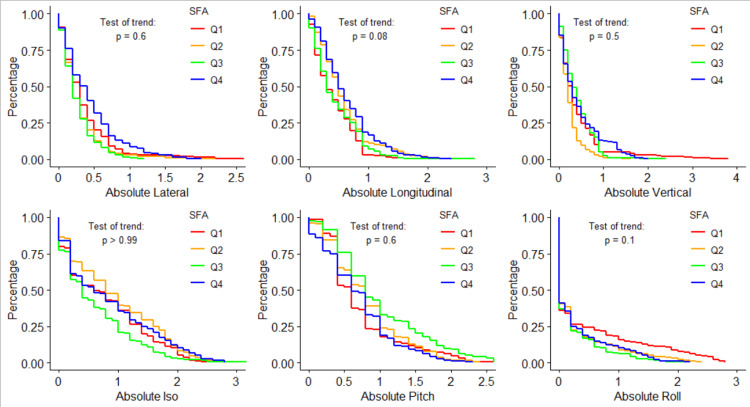
Shift probability curves for each shift direction and each SFA quartile, and the corresponding log-rank results testing for trends in shifts over different SFA quartiles SFA: subcutaneous fat area; Iso: yaw

Figure 3 similarly visualizes the log-rank test for each shift direction, but instead over the four SFA quartiles. Q1 of SFA is SFA$\leq$70, Q2 is 70<SFA$\leq$120, Q3 is 120<SFA$\leq$150, and Q4 is SFA>150. For all six tested shifts, none showed statistically significant trends over the four quartiles. This data also shows frequencies of longitudinal shifts greater than 10 mm as 2.1%, 8.7%, 9.0%, and 19.0% for each quartile, respectively.

**Figure 4:**
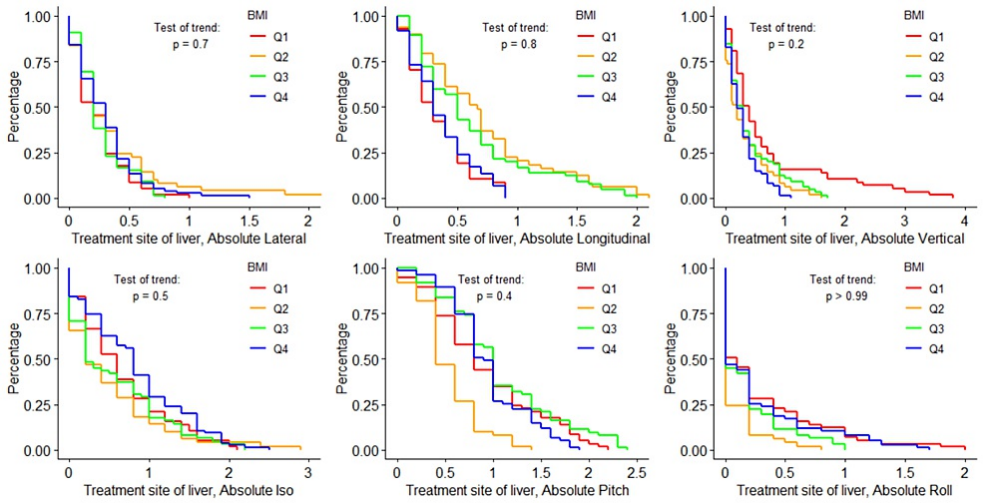
Shift probability curves for each shift direction and each BMI quartile, and the corresponding log-rank results testing for trends in shifts over different BMI quartiles for the liver treatment site subgroup BMI: body mass index; Iso: yaw

**Figure 5:**
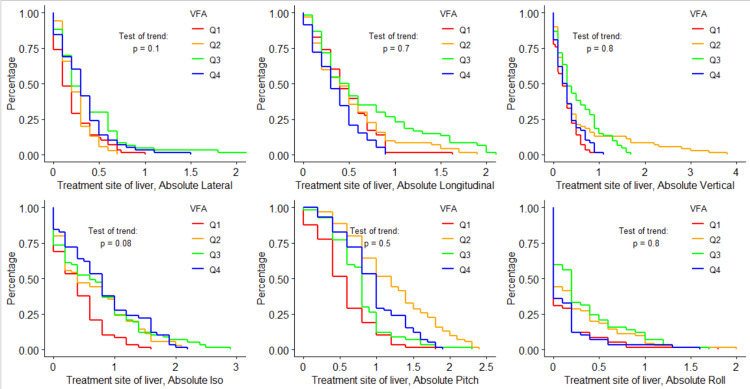
Shift probability curves for each shift direction and each VFA quartile, and the corresponding log-rank results testing for trends in shifts over different VFA quartiles for the liver treatment site subgroup VFA: visceral fat area; Iso: yaw

**Figure 6:**
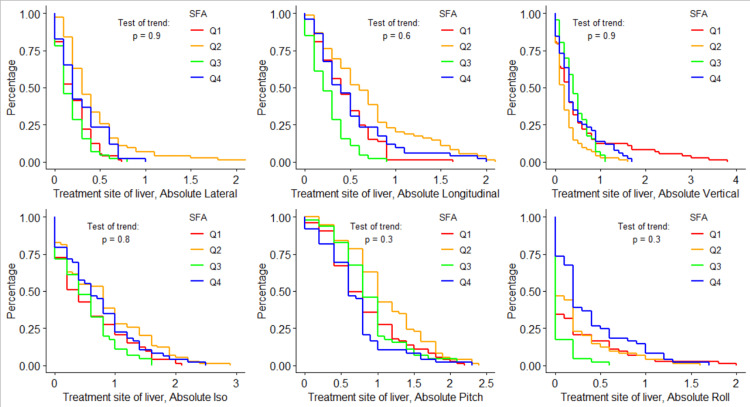
Shift probability curves for each shift direction and each SFA quartile, and the corresponding log-rank results testing for trends in shifts over different SFA quartiles for the liver treatment site subgroup SFA: subcutaneous fat area; Iso: yaw

Figures 4-6 visualize the log-rank test for each shift direction, using only data from the subgroup treatment site of the liver. For all six tested shifts, none showed statistically significant trends across BMI, VFA, or SFA quartiles.

Lastly, Table 3 shows the standard deviations of random setup errors (σ) for the upper quartile of each adiposity metric compared with the entire patient population.

**Table 3:** Standard deviations of the random setup error of all adiposity metrics in each shift axis as compared with the entire patient population VFA: visceral fat adiposity; SFA: subcutaneous fat adiposity; BMI: body mass index; σ: standard deviation of the random setup error

	σ Lat	σ Long	σ Vert	σ Yaw	σ Pitch	σ Roll
VFA high	0.12	0.12	0.08	0.18	0.14	0.12
SFA high	0.13	0.13	0.10	0.21	0.14	0.13
BMI high	0.13	0.13	0.09	0.18	0.14	0.14
All	0.06	0.06	0.05	0.11	0.08	0.08

## Discussion

Overall, the log-rank examination of shift probability suggests that abdominal RT patients with a larger BMI and greater visceral adiposity are statistically more likely to have larger shifts in the superior-inferior direction and larger pitch corrections relative to simulation. However, no statistically significant trends were seen for subcutaneous adiposity.

Interestingly, no statistically significant trends were observed for any adiposity metric for the liver patient subgroup. It is not completely clear why this is true, but it is possibly due to the location of tumors relative to surrounding adipose tissue. Liver tumors were some of the most superior targets included in this study, and the majority of abdominal adipose tissue, both subcutaneous and visceral, is located farther inferior. It is possible we may see similar trends for other anterior targets, such as the pancreas; however, we did not have enough patients in any other subgroup to appropriately characterize them.

Data from Zhao et al. suggested that (for thoracic tumors) shift errors were similar for low and moderate BMI patients, while large BMI patients showed significantly greater shift errors, particularly in the lateral direction [22]. Additionally, within their dataset, patients with a larger BMI showed a much larger percentage of shifts over 10 mm in all directions. Similar work done by Wong et al. specifically for patients being treated for prostate cancer also found that parameters such as BMI and subcutaneous adipose tissue thickness had a statistically significant correlation with shifts in the lateral direction, as well as an increase in the percentage of shifts greater than 10 mm [15].

While we did not see statistically significant trends for lateral shifts or a tangible increase in large shifts in the lateral direction, we did see increased lateral standard deviations of random error for the 4th quartile of all three adiposity metrics. In fact, similar to the results presented by Wong et al. [15], standard deviations of random error were larger for all axes regardless of adiposity metric, though those standard deviations were much lower in our study.

Similar to what was described by Wong et al., a common physical characteristic that could be contributing to these results is the size of the abdominal girth and its impact on the relative stability of setup tattoos. However, given that, in our institution and for all patients in this dataset, patient alignment in the longitudinal direction is done utilizing mainly the lateral tattoos, this does not completely account for the statistically significant trends in this axis. Also, while VFA showed greater statistical significance than SFA in relation to setup uncertainty, neither showed discernible advantages over BMI. Ultimately, the data suggest that larger adiposity, regardless of metric, is generally associated with more day-to-day variability in the left-right direction; however, visceral adiposity and BMI have a clearer relationship with large shift probabilities in the superior-inferior direction.

## Conclusions

Within this dataset, abdominal cancer patients showed statistically significant trends in shift probability as a function of BMI and VFA. Also, patients in the upper quartiles of all adiposity metrics showed increased standard deviations of random error in the lateral direction as well as an increase in shifts over 10 mm in the longitudinal direction. However, despite these relationships, neither VFA nor SFA offered discernible advantages in its relationship to shift uncertainty relative to BMI.
